# Sustainable Biomaterials: Current Trends, Challenges and Applications

**DOI:** 10.3390/molecules21010048

**Published:** 2015-12-30

**Authors:** Girish Kumar Gupta, Sudipta De, Ana Franco, Alina Mariana Balu, Rafael Luque

**Affiliations:** 1 Department of Pharmaceutical Chemistry, Maharishi Markandeshwar College of Pharmacy, Maharishi Markandeshwar University, Mullana, Ambala 133207, Haryana, India; girish_pharmacist92@rediffmail.com; 2 Departamento de Quimica Organica, Universidad de Cordoba, Campus de Rabanales, Edificio Marie Curie (C-3), Ctra Nnal IV-A, Km 396, E-14014 Cordoba, Spain; sudiptade22@gmail.com (S.D.); b12frloa@uco.es (A.F.); qo2balua@uco.es (A.M.B.)

**Keywords:** sustainable biomaterials, biomass precursors, waste valorization, porous carbons, bioplastics

## Abstract

Biomaterials and sustainable resources are two complementary terms supporting the development of new sustainable emerging processes. In this context, many interdisciplinary approaches including biomass waste valorization and proper usage of green technologies, *etc.*, were brought forward to tackle future challenges pertaining to declining fossil resources, energy conservation, and related environmental issues. The implementation of these approaches impels its potential effect on the economy of particular countries and also reduces unnecessary overburden on the environment. This contribution aims to provide an overview of some of the most recent trends, challenges, and applications in the field of biomaterials derived from sustainable resources.

## 1. Introduction

The interplay between biomaterials and renewable resources has provided a window of opportunity for the development of novel sustainable emerging strategies within the past few years. Humans used biomaterials in ancient times without actually knowing it. In recent times, a great deal of attention has been paid to their development from sustainable resources driven by the need to develop increasingly more sustainable alternatives to traditional materials. The urgent need for sustainable energy development depends on the advancements of green technologies and increasingly on biocompatible materials with properties comparable to existing materials because they are the way toward a more sustainable future.

## 2. Synthetic Routes: Towards Innovative Nanomaterials

The different techniques employed in the synthesis of biomaterials include thermal and/or hydrothermal synthesis or the use of surfactant-templates that allow a controllable design of (nano)material characteristics and properties [1]. The techniques generally extended have been based on a hard template, soft-template design, or a combination of both (even self-assembly in the absence of templates), which provided excellent fabrication protocols with various pros and cons as recently reviewed [2]. Nevertheless, a number of innovative promising methodologies has emerged in recent years. Hydrothermal carbonization (HTC), which takes place at moderate heating conditions and pressures in the presence of water, has been reported in the design of a number of different materials with relevant applications in fields such as drug delivery, catalyst, sensors, and CO_2_ sequestration [3,4,5]. Chemical Vapor Deposition (CVD) has been also reported in the synthesis of carbon nanotubes from various sources [6,7]. In the following sections, a range of bio(nano)materials will be disclosed in view of their synthetic protocols and properties as well as applications.

## 3. Carbonaceous Materials: Methods, Challenges, and Applications

In the context of sustainable biomaterial development, carbon is the most abundant element in the biosphere. Biomaterials derived from renewable carbon sources possess numerous applications in catalysis, electrochemical, photochemical, energy production, and polyester synthesis, *etc.* These have been reported to be obtained from a range of renewable resources including cellulosic fibers and agricultural biomass (such as corn stalks and pomelo skins) [8]. In an effort to address these challenges, this mini-review manuscript will briefly highlight the concept of valuable biomaterials derived from sustainable resources and the comparison in terms of their efficiency, usefulness, toxicity and eco-friendliness.

On the basis of previously reported findings, carbon resources could be summarized into several categories, such as: (i) graphitic nanostructures like carbon nanotubes, carbon nanofibers, graphene, graphene oxides, carbon nano-horns, onion like carbon, and graphene dots; (ii) carbon materials from deep eutectic solvents including choline chloride, mixtures of sugars, urea, and salts; (iii) Starbons derived from starch, alginic acid, pectin, chitosan, and carrageenan, *etc.*; (iv) hydrothermal and chiral carbon materials derived from biopolymers such as chitin, cellulose, oligosaccharides, and lignin.

A number of biomaterial and carbonaceous gels based on cellulose fibers, carbon nanotubes, and graphene have been successfully synthesized by different methods including freezing, thawing, hard and soft template methods, hydrothermal treatment, and freeze-drying. As an example to illustrate this concept, Zhao *et al.* recently explored the fabrication of cellulose carbon fibers with branched carbon nanotubes using pyrolysis. Branching was introduced by spreading a metal-containing salt solution on the surface prior to carbonization, which could effectively increase the specific surface area of carbon fibers and reduce its over potential for biochemical redox reactions [9]. Additionally, Wang *et al.* investigated the application of ^243^Am(III)-incorporated multiwalled carbon nanotubes for the removal of heavy metal ions from industry eluents in the direction of nuclear waste management [7]. In addition to these studies, Zhao *et al.* also demonstrated the role of graphene oxide nanosheets and its sulphonated version as biosorbents for the removal of Cd(II) or Co(II) ions, naphthalene, and 1-naphthol, respectively [10,11], setting up a nice starting point for the utilization of graphene nanomaterials for environmental remediation.

**Figure 1:**
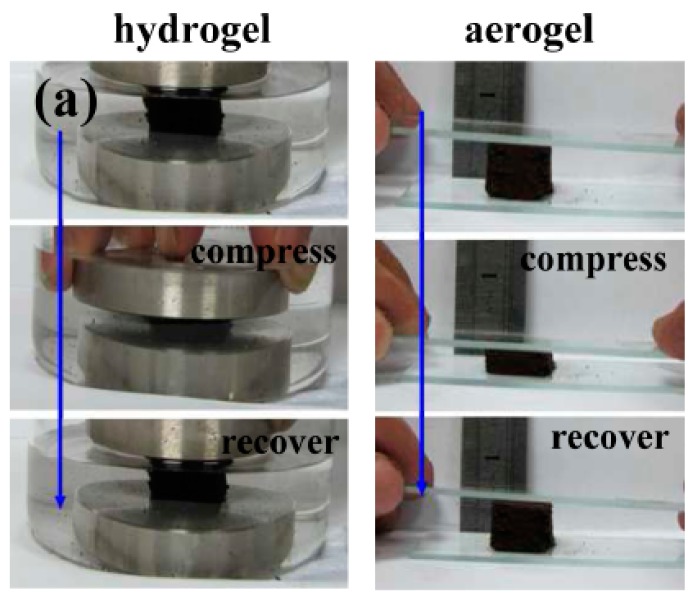
Digital photographs illustrating the compressive properties of hydrogels and aerogels.

Hydrothermal treatment attracted significant attention among listed approaches for biomaterials preparation as a green and inexpensive route for the preparation of carbonaceous gels. Hydrogels can be transformed into aerogels by replacing the liquid solvent in the hydrogels or other wet gels by air without collapsing the network assemblies (Figure 1). Conventional methods to prepare aerogels often involve templates (hard or soft), expensive chemicals, and supercritical drying. Moreover, the prepared aerogels are also associated with issues related to poor mechanical and/or thermal stability. To address these challenges, Wu *et al.* developed a simple and efficient template-free hydrothermal method to prepare low-cost 3-D carbonaceous flexible hydrogels and aerogels, and its fabrication by embedding Fe_3_O_4_ nanoparticles into the networks using crude biomass watermelon such as a carbon source [5]. These 3-D gels exhibited a high chemical activity and robust mechanical properties providing a remarkable potential as a scaffold to synthesize 3-D composite materials useful for electrochemical applications (improved transportation of both electrolyte ions and electrons).

Applications of this methodology have indeed enabled the prominent prospects to adsorbents, catalyst supports, supports for sensors, supercapacitors, and electrode materials for batteries and biomedical materials.

Porous carbons were also found to be very effective for dye removal and decoloration because of their large specific surface areas, pore volumes, chemical inertness, and good mechanical stability. Carbon materials fabricated from waste biomass have also shown promising applications as sorption materials, biochemicals, and others. As a key example, malachite green is a toxic dye component, and its discharge into aqueous media could pollute the aquatic life. Recently a study dealing with the adsorption of malachite green onto the sulphuric acid activated carbon prepared from naturally available waste biomass palm flower (Borassus aethiopum) has been reported and was found to be very effective [12]. In another report, banana peel was used to derive highly porous functional carbons featuring a high efficiency in the removal of methylene blue [13].

**Figure 2:**
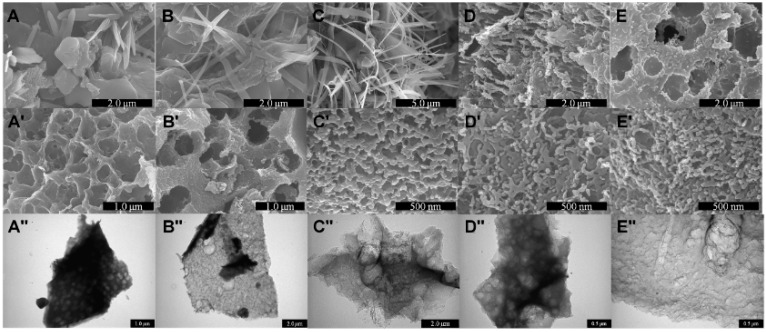
Effect of carbonization temperature on the formation or porous carbonaceous materials from human urine. SEM images of the mixture of carbon rock salts obtained at URC-X-BW, where X is (**A**) 700; (**B**) 800; (**C**) 900; (**D**) 1000, and (**E**) 1100 before washing with diluted HCl. Whereas (**A′**) SEM and (**A′′**) TEM images of URC-700; (**B′**) SEM and (**B′′**) TEM images of URC-800; (**C′**) SEM and (**C′′**) TEM images of URC-900; (**D′**) SEM and (**D′′**) TEM images of URC-1000; and (**E′**) SEM and (**E′′**) TEM images of URC-1100 after HCl washing, demonstrating the porous morphology of carbon obtained after the HCl treatment.

Carbonaceous biomaterials have also been synthesized from waste. A good example to illustrate this concept is the use of human urine (a polluting waste) to synthesize highly porous carbon materials containing heteroatoms (such as N, S, Si, and P) [14]. These materials offer a highly conductive hierarchical porous structure, which is responsible for excellent electrochemical properties (Figure 2).

Along the same lines, nanostructured carbonaceous materials—such as porous graphene-like nitrogen-doped carbon nanosheets from biomass/waste feedstocks (e.g., chitosan and urea) without any catalyst and post treatment—have been recognized as one of the most promising candidates for advanced energy storage applications [15]. Doping of various elements in porous carbonaceous materials can introduce the donor states near the Fermi level to generate n-type conductive materials, and the well-bonded miscellaneous element can drastically change the electronic and electrochemical performance by providing more active sites to increase the interaction between the carbon and adsorbents. For instance, organic-inorganic hybrid nanocomposites (such as different metal NPs-macroporous carbon systems) can be prepared by a facile two-step route consisting of carbonization and subsequent chemical synthesis or one-step carbonization [16]. These hybrid systems can provide good electrocatalytic performances toward the reduction of H_2_O_2_, the oxidation of glucose and amino acids. Heteroatom content, high porosity, improved surface area, and electrical conductivity are found to be the major factors governing the electrochemical activity.

Fermented rice is another highly abundant and inexpensive resource utilized as precursor for the preparation of porous carbons [17]. Nitrogen-doped carbon materials with high specific surface areas and high porosity can be obtained by a simple and scalable hydrothermal carbonization method. Compared to a commercial Pt/C catalyst, these nanocomposites can provide excellent electrocatalytic activity, better long-term stability, and methanol tolerance ability toward the oxygen reduction reaction (ORR), indicating a promising metal-free alternative to Pt-based cathode catalysts in alkaline fuel cells. In spite of several advantages, the majority of the traditional synthesis aspects had a negative impact on cost, environment, time, and complex routes at the industrial scale.

In context to the development of new technologies for H_2_ generation and CO_2_ capture and storage, many attempts have been made via the absorption process using alkanolamine solvents. However, this process demonstrated several disadvantages, such as high energy consumption, solvent regeneration, corrosion of the equipment, and toxicity. A lot of work has been done by a number of researchers to develop environmentally friendly strategies to provide an alternative to the valorization of wastes as a source to obtain novel engineering materials such as activated carbon or biomass carbon obtained from polysaccharides (starch and cellulose), biomass (sawdust) [18], birch wood xylan [19], and grasses as catalyst support [20]. In addition, these types of low-cost carbon materials can have extended applications as electrode materials and are adsorbent for color, odor, and hazardous pollutants, *etc.*

**Figure 3:**
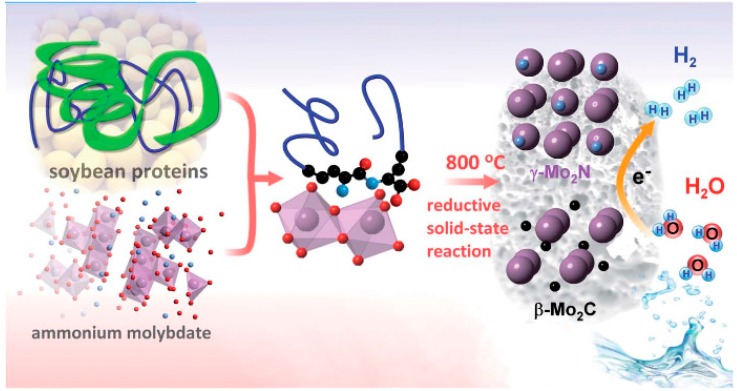
Design of a solid catalyst from soybeans and ammonium molybdate for electrochemical hydrogen production. A solid-state reaction between soybeans and the molybdate leads to their reductive carbonization and nitridation to form β-Mo_2_C and γ-Mo_2_N crystals. The structure of the amino moieties (blue balls) of soybean proteins is indicated. Plum balls: Mo, blue balls: N, black balls: C, and red balls: oxygen.

Hydrogen is a promising energy source and ideal from an environmental point of view as it can burn cleanly to produce water as the main combustion product. Out of several production methods, which include water electrolysis, steam reforming of natural gas, and coal gasification, water electrolysis is one of most attractive possibilities able to potentially provide domestically feasible, CO_2_ neutral, and non-polluting H_2_. The current challenge with the production of hydrogen is to reduce the use of noble metals or replace them with reasonably priced metal catalysts. In this context, there are mainly three strategies that have been adopted so far including: (1) optimization of the metal-hydrogen bond strength; (2) use of metal-coordination shells to amend electronic properties; and (3) surface nanostructuring to enhance the number and reactivity of catalytic sites. Several research groups have addressed various possibilities towards nonprecious metal based catalysts including molybdenum on graphene sheets obtained from soybeans (Figure 3) [21].

## 4. Biocompatible Nanocomposites

In another study, Jan *et al.* comparatively explored a simple, green, and versatile approach to use the cross-linked polypeptide hydrogels as templates for silica mineralization [22]. Those hybrid hydrogels were found to possess good cell compatibility and support cell attachment/proliferation with promising applications such as tissue engineering scaffolds. The compressive strength of the resulting silicified hydrogels, as well as the porosity of silica, can be simply controlled by tuning the polypeptide chain length and composition, which might influence its useful role for catalyst support, molecular sieving, and the preparation of various nanocomposites and biomaterials.

**Figure 4:**
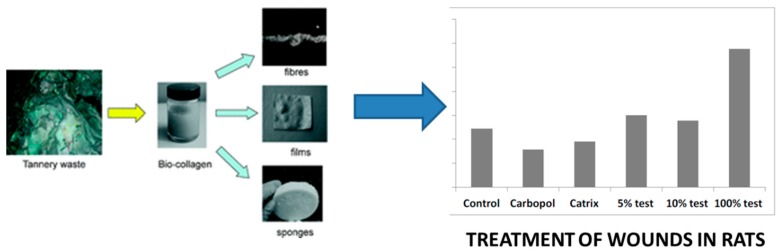
From slaughterhouse/tannery waste to biocollagenic polymers with applications in tissue regeneration. Reproduced by Permission of the Royal Society of Chemistry from reference [23].

More recently, the company Green Applied Solution Ltd. based in Spain (created with IP from our group) has been interested in developing various projects related to the use of biomass/waste types of feedstocks including collagenic-like biopolymers for the design of biocompatible nanocomposites. The extraction, isolation, and applications of biocollagen from slaughterhouse residues, as well as leather waste and useful materials, was previously explored by the group [23]. The utilized protocol involved a simple hydrolytic extraction process using acetic acid in which under optimized conditions of biocollagenic polymers of different molecular weight distributions could be obtained depending on the conditions. In general, these biocollagenic polymers could be formed into various morphologies including fibres, films, and sponges, with a relevant number of applications in tissue regeneration, biomedicine, and even cosmetics (Figure 4) [24].

In this regard, waste products with a high protein content (>80%) can be of interest for these experiments, not only related to the meat and leather industries, but also to production of collagenic products both in a gel-like phase (protein content 5%–10%) as well as in a solid form (with protein content over 20%). These may have interesting potential uses for various applications (e.g., amino acid solubilisation) as dietary supplements in cattle and even for health and care products.

Another interesting feature of hydrogels is their good compatibility with the living cells in the human body. This is one type of soft biomaterial that matches the stiffness of human tissues for tissue engineering and regeneration. Hydrogels are frequently employed for extracellular matrix functionalization and to provide appropriate mechanical cues. Hopkins and his co-workers investigated the mechanical and biochemical characteristics of silk hydrogels obtained from silk fibroin (cocoons of the Bombyx mori silkworm) for soft tissue engineering, specifically for the nervous system [25].

In context to the development of biodegradable products based on polysaccharides, Chang *et al.* demonstrated a method for the preparation of citric acid modified starch nanocomposite from pea starch [26]. Starch natively exists in granular form with few applications as compared to the plasticized form generally used for various purposes. Unfortunately, plasticized starch is usually sensitive to moisture, shows low tensile strength, and Young’s modulus. Those matrixes could not be gelatinized in hot water even at the high temperature and have been found potential in medical, agriculture, drug release, and packaging fields such as edible films, food packaging, and one-off packaging applications. A recent patent also discloses the usefulness of nanocomposite biomaterials of nanocrystalline cellulose and polylactic acid [27].

### 4.1. Silicate-Type Materials from Biomass Residues

Rice husk and rice straw are two of the most important by-products of rice cultivation and processing. The global rice production increases at an average of 16 million tons per year [28]. Rice by-products have essentially different compositions but are similar in terms of different present fractions, namely cellulose (30%–40%), hemicelluloses (20%–30%), lignin (10%–15%) and ash (8%–20%) [29]. The metal content in rice husks is of particular interest as it is very much enriched in silica (SiO_2_) that can be potentially extracted from rice husks using alkali solutions. The proposed approach has been previously attempted utilizing waste ashes from a commercial biomass combustion facility in the UK [30] as well as related ash-type waste feedstocks [31,32]. The methodology involved a simple hydrothermal extraction with KOH to generate potassium silicate solutions under reproducible 60%–70% extraction yields, which were subsequently analyzed and validated using a straightforward infrared method for simple quantification [30]. Upon extraction, the alkali silicate solutions were used as a silica source for the preparation of high surface area mesoporous silica materials of typical MCM-41 structure by the addition of a structure directing agent (cetyltrimethylammonium bromide, CTAB) and similar synthetic conditions to those generally employed for the preparation of such mesoporous silicate materials.

**Figure 5:**
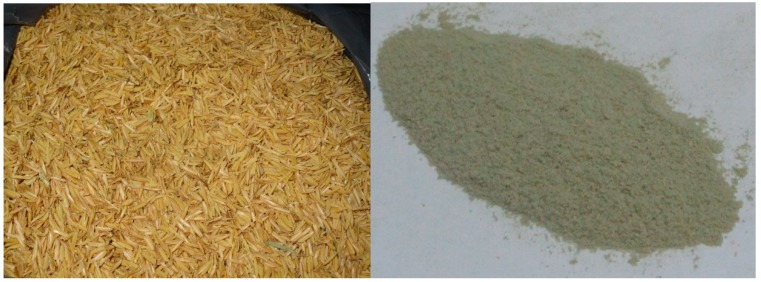
Pretreatment of rice husk (**left image**) to fine sieved powder (**right image**) via milling prior to processing.

Based on the aforementioned previous work, an analogous system has been developed for the production of different high added value materials, chemicals from rice husks, and more recently, ashes from the combustion of rice husks. The composition of rice husks are remarkably more complex as compared to that of waste ashes (>90% metal oxides with an over 80% silica content). Hence, preliminary experiments were aimed to find out more or less optimum conditions to produce valuable compounds from rice husk. The starting material is generally milled in a planetary ball mill to a fine powder, trying to minimize the large volume of rice husk for a simplified and more efficient processing (Figure 5).

Under optimized conditions, including extraction, work-out, and treatment of the first developed protocol can yield a highly pure and crystalline silicate material with about a 20% yield (by dry weight) using a HCl-mediated extraction under microwave irradiation.

### 4.2. Advanced Nanocomposites and Nanomaterials from Natural Waste

Tobacco waste is also an important issue in both developed and developing countries. For example, in China, tobacco sales leave millions of tons of tobacco waste yearly that find little use aside from disposal/landfilling and burning. In collaboration with the School of Food and Biological Engineering at Zhengzhou University of Light Industry (Prof. Chunping Xu), the company has found an interesting number of applications of bio-engineered polysaccharides from a simple but highly effective aqueous extraction from tobacco [33], which more recently also extended to the utilization of tobacco waste. In their proposed approach, different sources of tobacco were utilized including Burley (cured) and flue-cured. The most extended practice for the extraction and separation of polysaccharides from tobacco leaves was a simple aqueous extraction process assisted by ultrasounds [33]. Upon extraction, polysaccharides were subsequently separated and isolated using Sepharose-type columns and eventually characterized and quantified. Several fractions of different polysaccharide extracts could be isolated from tobacco leaves with interesting biological activities [33].

These extracts were mainly tested for antioxidant activities, with results pointing to excellent radical scavenging properties (for both hydroxyl as well as DPPH) in a dose-dependent manner [33]. In some other cases, analogous polysaccharides extracted from tobacco or other biomass-derived sources have been reported to have promising antimicrobial and neuroprotective activities [34].

In a similar approach, exopolysaccharides (EPS) can also be produced via fermentation of certain organisms [35,36]. The group of Xu *et al.* has extensively worked with exopolysaccharides produced by submerged cultures of fungi including *Boletus aereus* [35] and *Trametes gibbosa* [36]. Importantly, EPS generated upon fermentation exhibited remarkable biological properties both *in vitro* and *in vivo*, ranging from antioxidant (e.g., radical scavenging) [35], antimicrobial (due to the presence of 2-hydroxy-6-methylbenzoic acid), and antitumoral to the most interesting hypoglycemic and hypolipidemic effects [36]. EPS hypoglycemic and hypolipidemic effects were investigated in streptozotocin-induced diabetic rats, including a decrease in plasma concentrations of glucose (17%–37%), total cholesterol (14%–26%), and triacylglycerol (12%–24%) as well as aspartate aminotransferase activity (up to 20%) [36].

Exopolysaccharides were produced from batch lab fermentations (1–15 L [35,36]), although successfully scaling up for the production of mycellial extracellular products was proved in large scale industrial fermenters (2000 L) from the *Ophiocordyceps sinensis* strain Cs-HK1 [36]. Apart from the aforementioned antimicrobial, antitumoral, antioxidant, hypoglycemia, and hypolipidemia activities of reported EPS, the production of important chemical compounds (e.g., betulin, 2-hydroxy-6-methylbenzoic acid, *etc.*) has also been reported [36]. These can offer additional possibilities for further developments in important market products for antioxidant, antimicrobial, and ultimate sportive formulations for athletes as well as antitumoral and cytotoxicity protection.

In view of the relevant properties and biological activities of these extracts and exopolysaccharides, advanced applications for extracted polysaccharides and related compounds from tobacco as well as from other sources (e.g., macroalgae) were envisaged [37].

**Figure 6:**
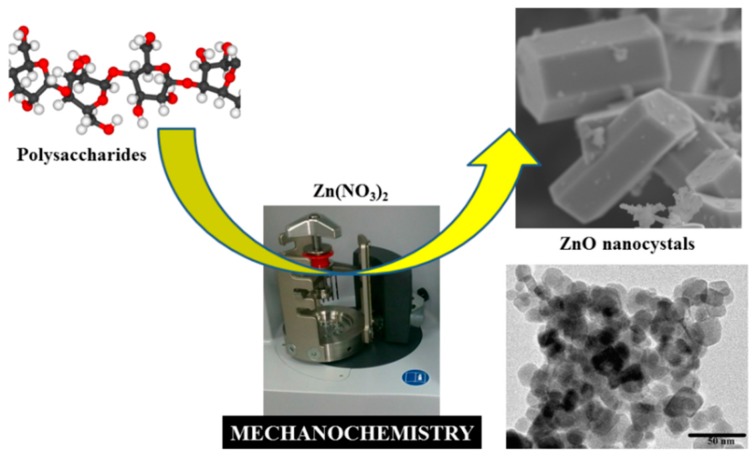
Mechanochemical production of designer nanomaterials from various polysaccharides.

**Figure 7:**
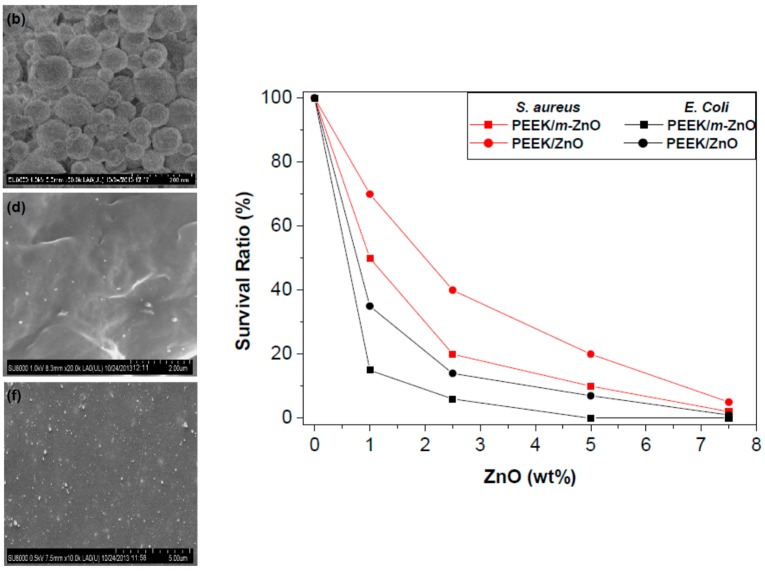
SEM of synthesized novel poly(ether ether ketone)/ZnO nanocomposites (**b**, **d**, **f images**) with biotemplated ZnO for antimicrobial applications for *S. aureus* and *E. Coli*. Reproduced by permission of the Royal Society of Chemistry from Reference [39].

The first initiative related to the utilization of such polysaccharides as sacrificial templates for the production of a wide range of nanomaterials including nanocrystals of metal oxides. Preliminary research results from the group indicated that the utilization of pure polysaccharides including starch and alginic acid as templates in a dry ball milling methodology could lead to advanced nanocrystals of metal oxides (ZnO) in high purities and with a highly crystalline nature (Figure 6) [38]. Importantly, the synthesized porous ZnO nanostructures were found to have excelling photocatalytic properties for the degradation of pollutants (e.g., phenols) in water [38] as well as very promising antimicrobial activities when utilized as formulations in polymer nanocomposites (Figure 7) [39] and antitumoral activities with unprecedented toxicities for human cell lines [40].

These results were initial proof-of-concept from pure polysaccharides but in some cases algae-extracted polysaccharides show a remarkable potential as replacement of pure compounds in the proposed technologies [38].

## 5. Perspectives on Sustainable Biomaterials

The development of economic and environmentally benign processes for the scale-up production of materials, chemicals, and fuels is one of the challenges for the 21st century. Selected examples for bio(nano)material designs in view of their future applications clearly illustrates the potential of such bio-derived materials including carbonaceous materials and biocompatible nano-composites from natural sources in a wide range of applications. The use of low cost and alternative renewable precursors (*i.e*., biomass and residues) to produce controllable and well defined nanostructures is the way forward in this regard. In some cases, the challenge lies in the possibility to carefully control the properties of the synthesized biomaterials (due to the complexity of the starting material, presence of impurities, *etc.*), but these issues have been occasionally circumvented in cases depending on the biomaterial synthetic protocol and/or the selected future application. As an example, the presence of heteroatoms including Nitrogen or Boron can improve the electrochemical performance. In some other cases, the formation of nanocomposites or the presence of another substrate/starting material (e.g., graphene) can lead to advanced materials with high-end applications such as electrodes in energy storage devices (batteries, fuel cells, *etc.*) where physical properties like conductivity, flexibility, transparency, and mechanical strength are compulsorily needed. These biomaterials with customized composition and porosity can be suitable as electrodes in supercapacitor cells as well as adsorbents for CO_2_ sequestration. From personal experience, a deep knowledge in the structure and composition of the starting material (e.g., lignocellulosic fractions) is key to lead future advances in the field of sustainable biomaterials for different applications.

Furthermore, the search for alternative and *benign by design* protocols is able to provide a similar performance to traditional methods is another area of future interest. Many traditional methods involved complex and tedious protocols with poor control over final particle size, unsolved or un-predictive mechanistic effects of temperature, pressure, and pH on the biomaterials morphology, mechanical, or thermal stability, *etc.* There are still many challenges to overcome in the sustainable design of biomaterials including reproducibility, high porosity, controllable properties, and stability, *etc.*, which need to be addressed. Nevertheless, the production of nanocomposites and nanomaterials from polysaccharides including starch, alginic acid, and exopolysaccharides obtained from natural products like tobacco or macroalgae clearly illustrate the potential of such *benign by design* concepts. Further research is currently ongoing in this area with future developments to be reported in due course to address the daunting challenges of resource scarcity and energy demands for the future generations.

## 6. Conclusions

A number of developments on a series of protocols and technologies to valorize waste/biomass into highly valuable biomaterials and bio-products were highlighted in this contribution. From biomass/waste valorization to biomaterials with different applications, the present contributions illustrate the potential for a range of nanomaterials in catalysis, adsorption, environmental remediation, energy conversion, and storage, as well as biomedicine. Until now, only a few significant efforts have been dedicated to the improvement of some of the highlighted (nano)structures, therefore technologies and chemistries still require a significant amount of work in the future. In any case, innovative and emerging green technologies for the design of biomaterials can lead the way toward an economical and sustainable society for the betterment of mankind as illustrated by the key examples included in this contribution, which we sincerely hope can stimulate further work within the scientific community.
